# Real-world evidence for Pompe disease remains fragmented. Comment on “A rare partnership: patient community and industry collaboration to shape the impact of real-world evidence on the rare disease ecosystem” by Klein et al.

**DOI:** 10.1186/s13023-025-03552-3

**Published:** 2025-02-14

**Authors:** Michelle E. Kruijshaar, Tiffany House, Benedikt Schoser, Pascal Laforêt, Maudy T. M. Theunissen, Stephan Wenninger, Thomas Hundsberger, Jordi Diaz-Manera, Ans T. van der Ploeg, Nadine A. M. E. van der Beek

**Affiliations:** 1 Center for Lysosomal and Metabolic Diseases, Department of Pediatrics, Erasmus MC, University Medical Center, Rotterdam, The Netherlands; 2 International Pompe Association, Baarn, The Netherlands; 3 Acid Maltase Deficiency Association (AMDA), San Antonio, TX USA; 4 Department of Neurology, Friedrich-Baur-Institute, LMU Clinics Munich, Munich, Germany; 5 Neurology Department, Raymond Poincaré University Hospital, Paris, France; 6 North-East-Ile-de-France Neuromuscular Reference Center, APHP and FHU PHENIX, Paris, France; 7 Université Versailles-Saint Quentin, Paris-Saclay, Paris, France; 8 Center for Lysosomal and Metabolic Diseases, Department of Neurology, Erasmus MC, University Medical Center, Rotterdam, The Netherlands; 9 Department of Neurology, Cantonal Hospital St Gallen, St Gallen, Switzerland; 10 John Walton Muscular Dystrophy Research Center, Newcastle University & Translational and Clinical Research Institute, Newcastle Upon Tyne, UK

**Keywords:** Rare disease registries, Real-world evidence, Pompe disease, Patient-reported outcomes

## Abstract

In a recent publication by Klein et al., the need for real-world data on rare diseases is highlighted. We strongly support this need, and the collaboration with the patient community to collect data, as promoted in this publication. Our concern, however, is that this paper may be misunderstood as suggesting that the Sanofi-run Rare Disease Registries (RDRs) are sufficient to provide the datasets needed to evaluate current and future therapies. Industry-driven registries focus on their own product(s) and, therefore, do not provide the opportunity to compare products from different companies. Today, multiple companies produce treatments for all diseases included in the RDRs. Each company will have to run its own registry for regulatory purposes. This will lead to data fragmentation, which is prohibitive of truly understanding the effects of the various treatment options for these rare diseases. Therefore, independently funded and owned registries are essential to generate real-world evidence (RWE) unrelated to specific products. We discuss options for this for Pompe disease, including the International Pompe Survey, which has collected patient-reported outcomes independently from industry since 2002. This letter aims to raise awareness of the problem of siloed data and advocate for a new way forward where independent registries provide post-marketing surveillance data.

The recent publication by Klein et al. in this journal [1] underlines the importance of collecting real-world evidence (RWE) for rare diseases and the crucial role the patient community plays in maximising the value of RWE. Lysosomal storage diseases (LSDs) are a group of rare, inherited metabolic disorders in which deficiencies in lysosomal enzymes result in the accumulation of substrates in the lysosomes of cells, causing problems in various organs, such as growth problems, muscle weakness, and mental development. LSDs and other rare diseases are difficult to study due to the small number of patients available. Klein et al. describe a group of registries set up by Sanofi to monitor patients with four different LSDs (Gaucher, Fabry, MPS I and Pompe disease), for which they produce enzyme replacement therapy (ERT). They argue that registries in general - and their registry in particular - can provide critical, long-term data needed to inform all key stakeholders. The paper also highlights how the contribution of their registries has been enhanced by collaborating with the patient community.

While we greatly applaud the emphasis on collecting real-world data through registries and the vital role of the patient community in this endeavour, it is important to point out that the success of an industry-led registry in providing information about patients from different backgrounds and severities that is useful to *all* stakeholders is only possible when this industry is the sole producer of treatment for this disease. When there is only one treatment producer, data can be collected and successfully harmonized to show RWE of treatment as the only alternative to that treatment is the natural history of the disease.

We are now at a crucial point in time where second-generation ERTs and other innovative treatments for LSDs are being developed by multiple companies. As it stands, all of these companies will be required to set up registries for the diseases that they are involved with to comply with post-marketing surveillance requirements from regulators such as the US Food and Drug Administration and the European Medicines Agency. With more than one industry-driven registry for each disease, data will become fragmented and siloed (split into isolated parts), as clinicians and patients cannot be expected to enter the same information into multiple registries. In addition to the issue of multiple industry-driven registries per disease, these registries are not easily accessible for broader research, despite claims to the contrary by Klein et al., as interested research groups cannot easily gain access to the datasets.

For Pompe disease, the International Pompe Association (IPA), the European Pompe Consortium (EPOC), and other expert clinicians worldwide have, therefore, been discussing ways to consolidate international data independently of industry initiatives. Different options for collating physician-reported outcomes all come with their own challenges. Setting up a new registry, like the patient-driven registry for Niemann-Pick Disease [2], for example, is hindered by concerns about having to enter data twice (into both the new registry and the industry-driven one(s)) [3]. A few successful research projects have brought together data from clinical centres in different countries [4, 5], but this process is highly time-consuming and thus cannot provide timely data to support public health and regulatory decisions. At the national level, France and Switzerland have reached agreements for industry-independent registries to collate their data, which can then provide reports for industry [6]. However, such initiatives remain the exception rather than the rule. To achieve sufficient patient numbers for drawing robust conclusions, international agreement is needed on how to consolidate physician-reported outcomes. This effort must include clinicians, regulators, and industry stakeholders.

The same is needed for patient-reported outcomes (PROs). For Pompe disease these have been collected internationally, independent of industry, for many years. The International Pompe Survey, an annual patient-reported survey from patients worldwide, has been conducted by the IPA and the Erasmus Medical Center since 2002. It collects data from late-onset Pompe patients aged 16 and over. Reaching out directly to patients enables access to large numbers of patients receiving diverse treatments worldwide [7]. The PROs that are collected this way can provide valuable insights into both the natural history of the disease and the effects of treatment [7–19]. PROs evaluate diverse aspects including health-related quality of life, participation in daily life, fatigue, and wheelchair use. By tracing patients lost to follow-up, the survey has also generated survival data, which have proven crucial in reimbursement discussions for late-onset patients.

The ability of the International Pompe Survey to serve as an independent source of RWE on PROs in the future, in addition to stakeholder support, depends on its capacity to meet diverse data needs, including reports for industry and regulators as well as data on children and patients with classic-infantile Pompe disease. As Klein et al. announced, the RDR is now also rolling out similar PRO questionnaires to patients. This means that the International Pompe Survey will now compete with the Sanofi-driven registry for patients’ time, much like how multiple industry-driven registries compete for clinicians’ time. As a result, none of these datasets will provide a complete picture of the international Pompe population going forward.

In conclusion, we believe that the only solution is to move away from the primary cause of fragmented data: industry-owned registries. In a multi-treatment space, ideally, no individual company, institution, or otherwise restrictive group should collect siloed data. Instead, data collection should be managed by an industry-independent body, operating under the FAIR data principles, with international support from all clinicians, patients, and regulatory agencies.

## Data Availability

We do not analyse or generate any datasets for this publication, as this letter discusses different data sources and how they are collected.
